# Regulatory T Cells Suppress Effector T Cell Proliferation by Limiting Division Destiny

**DOI:** 10.3389/fimmu.2018.02461

**Published:** 2018-10-30

**Authors:** Mark R. Dowling, Andrey Kan, Susanne Heinzel, Julia M. Marchingo, Philip D. Hodgkin, Edwin D. Hawkins

**Affiliations:** ^1^Immunology Division, The Walter and Eliza Hall Institute of Medical Research, Parkville, VIC, Australia; ^2^Department of Medical Biology, The University of Melbourne, Parkville, VIC, Australia

**Keywords:** T cells, regulatory T cells (Tregs), modeling and simulation, cytokines, immunity

## Abstract

Understanding how the strength of an effector T cell response is regulated is a fundamental problem in immunology with implications for immunity to pathogens, autoimmunity, and immunotherapy. The initial magnitude of the T cell response is determined by the sum of independent signals from antigen, co-stimulation and cytokines. By applying quantitative methods, the contribution of each signal to the number of divisions T cells undergo (division destiny) can be measured, and the resultant exponential increase in response magnitude accurately calculated. CD4^+^CD25^+^Foxp3^+^ regulatory T cells suppress self-reactive T cell responses and limit pathogen-directed immune responses before bystander damage occurs. Using a quantitative modeling framework to measure T cell signal integration and response, we show that Tregs modulate division destiny, rather than directly increasing the rate of death or delaying interdivision times. The quantitative effect of Tregs could be mimicked by modulating the availability of stimulatory co-stimuli and cytokines or through the addition of inhibitory signals. Thus, our analysis illustrates the primary effect of Tregs on the magnitude of effector T cell responses is mediated by modifying division destiny of responding cell populations.

## Introduction

CD4^+^CD25^+^Foxp3^+^ regulatory T cells (Tregs) play a critical role in immune homeostasis. However, the precise mechanism of regulatory function on effector T cells remains contentious. Important roles for modulation of co-stimulation by dendritic cells (1–4), absorption of cytokines such as IL-2 (5–8), secretion of inhibitory cytokines such as TGF-β, IL-10 and IL-35 (9–13) and direct cell-contact dependent mechanisms (9, 14) have all been demonstrated in a variety of *in vitro* and *in vivo* systems (15–18). The relative quantitative importance of these different mechanisms is unknown and may depend on context. Apart from suppressing proliferation, Tregs are also known to modulate the function of effector T cells. For example, Maeda et al. recently showed that Tregs can induce self-reactive human CD8^+^ T cells (Melanin-A specific) to adopt a CCR7^+^CTLA-4^+^ anergic phenotype in response to peptide stimulation *in vitro*, as well as reducing their proliferation via modulation of dendritic cell co-stimulation (19).

Recent work by Marchingo et al. has defined a quantitative framework for understanding signal integration by T cells (20). A key concept is the notion of “division destiny”—the number of divisions a cell undergoes before ceasing proliferation and reverting to a quiescent state, first described in B cells (21–23). The mean division destiny of CD8^+^ T cells was shown to be the linear sum of independent contributions from antigen, co-stimulation and cytokines, allowing quantitative prediction of the magnitude of the T cell response from knowledge of the individual stimuli. Heinzel et al. subsequently demonstrated that this quantitative signal integration to determine division destiny can be inferred by levels of Myc within T cells and B cells, providing a molecular mechanism for this phenomenon (24).

We tested whether the calculus of division destiny could be used to quantify the action of Tregs during suppression of effector T cell proliferation. We hypothesized that Tregs may potentially function in an opposing mechanism to T cell co-stimulation, and thus manifest suppression of effector T cell proliferation via a reduction in division destiny in the effector T cell population. Here, using quantitative methods, we illustrate that the dominant action of Tregs is through “subtracting” division destiny in responding T cells in a dose-dependent manner, in comparison to inducing more rapid death or slowing proliferation. These results provide a quantitative framework for studying different mechanisms of suppression in immune responses including genetic polymorphisms associated with autoimmunity or inflammation. Furthermore, they highlight that division destiny is a universal cellular parameter central to not only positive regulation of immune responses, but also effector response suppression.

## Materials and methods

### Mice

All experiments were performed using C57BL/6 mice bred and maintained under specific pathogen-free conditions in the Walter and Eliza Hall Institute (WEHI) animal facilities (Parkville, Victoria, Australia) and used between 6 and 12 weeks of age. All experiments were performed under the approval of the WEHI Animal Ethics Committee.

### CD4+CD25+ treg and CD4+CD25-CD62L+ teff cell purification

CD4^+^CD25^−^CD62L^+^ effector T cells (Teff) were isolated from pooled mouse lymph nodes (inguinal, axillary, brachial, superficial cervical, and lumbar) and spleens by negative and positive selection using the mouse naïve CD4+ T cell isolation kit (Miltenyi). CD4^+^CD25^+^ Tregs were prepared from pooled spleen and total lymph nodes (inguinal, axillary, brachial, superficial cervical, and lumbar) of C57BL/6 mice. Cell suspensions were stained with anti-CD4^−^PerCP-Cy5.5, anti-CD25-FITC, and enriched for CD25^+^ cells using anti-FITC beads (Miltenyi). Cells were then sorted for CD4^+^ CD25^hi^ on a BD FACSAria. Treg purity was checked using intracellular staining for Foxp3 and in all experiments was >90%. Irradiated splenocytes were prepared by red cell lysis of whole spleen suspension and irradiated at 3,000Gy.

### Celltrace oregon green labeling

For division tracking, Teffs were labeled with a final concentration of 20 μM Cell Trace Oregon Green (Invitrogen) by incubation for 10 min at 37°C at a cell density of 10^7^ cells/mL in phosphate-buffered saline (PBS) with 10% bovine-serum albumin (BSA).

### Cell culture

Cells were cultured in RPMI 1640 medium (Invitrogen) supplemented with non-essential amino acids, 1 mM Sodium-pyruvate, 10 mM HEPES, 100 U/mL Penicillin, 100 μg/mL Streptomycin (all Invitrogen), 50 μM 2-mercaptoethanol, 2 mM L-glutamine (both Sigma) and 10% FCS (JRH Biosciences and Invitrogen). Cells were incubated in a humidified environment at 37°C in 5% CO_2_.

The *in vitro* Treg suppression assay was set-up as follows (25). Twenty thousand Teffs were co-cultured with 80,000 irradiated splenocytes and 2 μg/mL anti-CD3 (clone 2C11, WEHI antibody facility, Australia) and a varying ratio of Tregs. Proliferation was analyzed by flow cytometry for the next 4 days.

For experiments mimicking suppression the following reagents were added to cultures: CTLA4-Ig (prepared from COS cells, provided by Peter Lane), anti-mouse IL-2 monoclonal antibody (purified from hybridoma cell line S4B6, WEHI), TGF-β (eBioscience), recombinant murine IL-10 (purified from baculovirus-transfected Sf21 insect cell supernatant, DNAX).

### Flow cytometry analysis

Triplicate wells were harvested at each time point after addition of a known number of CaliBRITE microbeads (BD) to facilitate quantification of absolute cell numbers. Cells were analyzed on a BD FACSCanto.

### BrdU labelling

Detection of intracellular BrdU was performed using a BrdU staining kit (BD Pharmingen) as per manufacturer instructions.

### Calculation of cell numbers per division, cohort number and mean division number of dividing cells

The number of cells per division, *n*_*i*_, *i* = 0, 1, …, 7, 8^+^, was determined by flow cytometry with gating for 2-fold dilution of Cell Trace Oregon Green intensity and the ratio of analyzed cells to the known number of microbeads (division number >7 could not be resolved above background autofluorescence, and 8^+^ refers to all cells gated as having divided 8 or more times).

The number of undivided cells is *n*_0_, and the number of dividing cells is:

(1) $$\begin{array}{r} N_{div}=\overset{8+}{\underset{i=1}{\sum }}n_{i} \end{array}$$

Following (26), the precursor cohort numbers for each division, *c*_*i*_, were calculated by dividing the cell number per division by two to the power of division number, in order to remove the expected expansion of cell number with division in the absence of death:

(2) $$\begin{array}{r} c_{i}=\frac{n_{i}}{2^{i}} \end{array}$$

The total cohort number, *C*, is the sum of the cohort numbers over all divisions:

(3) $$\begin{array}{r} C=\overset{8^{+}}{\underset{i=0}{\sum }}c_{i} \end{array}$$

The cohort number would remain equal to the starting cell number if there were no cell death in the system, and therefore comparison of differences in cohort number over time according to a varying condition can be used to identify effects on survival (20, 24, 26–28).

Plots of mean division number against harvest time can be used to estimate proliferation features, including average time to first division, subsequent division rate and division destiny (20, 26, 28, 29). A number of methods have been used to calculate mean division number. Here, as not all anti-CD3 stimulated T cells enter division, we averaged the dividing cells only. This value, mean division number of dividing cells (MDN_div_), is calculated as:

(4) $$\begin{array}{r} MDN_{div}=\frac{\sum_{i=1}^{8^{+}}ic_{i}}{\sum_{i=1}^{8^{+}}c_{i}} \end{array}$$

A plateau in MDN_div_ can indicate that the cells have stopped dividing having reached their division destiny.

## Results

### Regulatory T cells do not reduce survival or activation of effector T cells *in vitro*

In principle, regulatory T cells may suppress effector T cells by directly inducing death, by reducing activation and recruitment into division, by slowing the division rate, or by reducing division destiny. To decipher the effects on these different parameters, we analyzed an *in vitro* suppression assay using the established precursor cohort method (26, 29). This approach uses quantitative graph-based methods to track the fate of founder cells seeded in culture during *in vitro* proliferation assays and allocate effects to changes in division rate, division destiny or overall cell survival. We designed our experimental approach using a suppression assay that reflects the majority of assays used in studies of Treg biology. Teffs labeled with the division tracking dye Cell Trace Oregon Green were co-cultured with varying ratios of Tregs, irradiated splenocytes as antigen-presenting cells (APCs), and anti-CD3 as a polyclonal T-cell-receptor stimulus (25). Addition of counting beads at the time of harvest allowed quantification of absolute cell numbers per division.

**Figure 1:**
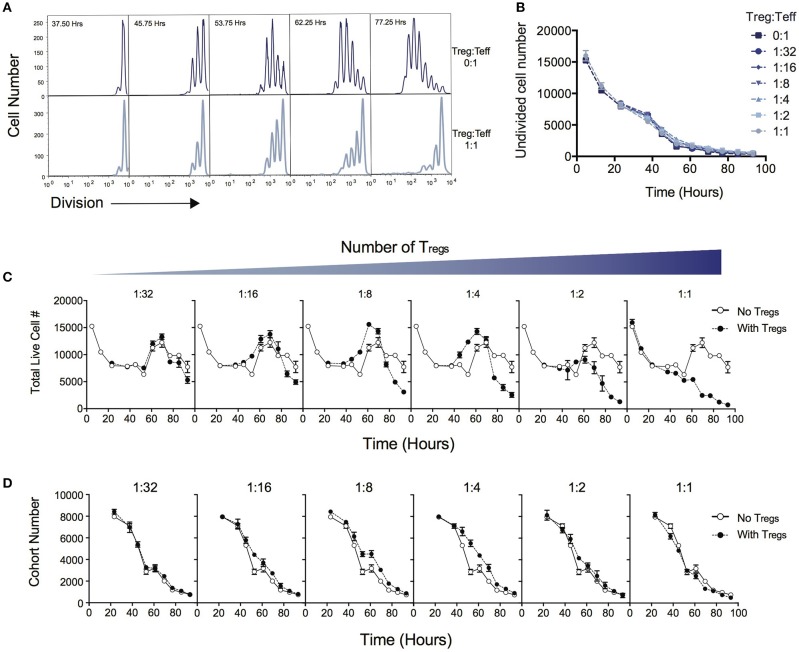
Quantitative analysis of the effect of Tregs on Teff stimulation. CD4^+^CD25^−^CD62L^+^ effector T cells (Teffs) labeled with cell division dyes were co-cultured with varying ratios of Tregs and the response measured. **(A)** Example timecourse of cell division progression in cultures without Tregs (top panel) and with Tregs (lower panel). **(B)** Number of undivided Teff cells in culture over time. Data shown are mean +/– SEM of triplicate samples. One representative data set from three independent experiments is shown. **(C)** The effect of Tregs on the total live cell number of Teffs over time for varying ratios of suppression is shown. For each graph, the Treg ratio (closed circles) is overlaid with the control culture with no Tregs added (open circles). **(D)** The cohort number over time of Teff cells is shown for varying ratios of Tregs. For each graph, the Treg ratio (closed circles) is overlaid with the control culture with no Tregs added (open circles).

Figure 1A demonstrates the suppressive effect of Tregs on division of Teff over the time course of T cell stimulation as measured by dilution of cell division tracking dyes. When two ends of the spectrum are compared (no Tregs vs. a high Treg:Teff ratio), the progression through division of the Teff population is significantly reduced. In this system not all T cells are activated to enter division, and cells that are not activated display different survival kinetics than activated cells (27, 30). We first asked whether the suppressive effect of Tregs could be ascribed to a reduction in either the survival of undivided cells or in the proportion of cells induced to divide, as either conclusion could be reached by comparing division profiles shown in Figure 1A. Either of these processes would affect the number of undivided cells measured in culture over time. Figure 1B shows that the number of undivided cells is unaffected by the Treg ratio over the course of the experiment. Thus, contrary to the above expectation, survival of undivided cells and recruitment into division is not affected by Tregs, and an alternate explanation must be sought.

Next, we examined total cell numbers in culture. Figure 1C quantifies the response of Teffs in culture over time as represented by total cell numbers in the context of varying the Treg ratio. The peak of the response was ~60 to 70 h post stimulation for all Treg ratios, followed by a decline thereafter. Late in the culture, after 70 h, the highest cell numbers were observed in the absence of Tregs, and the addition of Tregs reduced the Teff number in a dose-dependent manner, as expected. Interestingly, between ~40 and 60 h we noted an increase in cell number at intermediate ratios of Tregs (1:16, 1:8, 1:4), compared with lower or higher ratios of Tregs, which was unexpected and did not correlate with the overall trend seen in cell numbers at the end of the experiment.

We investigated how the Teff response was altered in the presence of increasing numbers of Tregs by applying the precursor cohort method (20, 26, 27). As described in Methods, the cohort number is defined as the sum of the cell numbers in each division divided by two to the power of division number. Calculating the cohort number removes the effect of cell division on cell number, allowing an analysis of survival of the original cohort of cells placed in culture, independently of other kinetic changes. Figure 1D illustrates the effect of Tregs on the cohort number over time. In general, increasing numbers of Tregs did not induce a more rapid decline in the cohort numbers over time, indicating the mechanism of suppression is not via active induction of death of Teffs. This result is also supported by observing survival early in the culture, prior to entry into first division (< 50 h—Figure 1C), where Teffs appeared to die at a rate that was independent of Treg ratio. The exception is that we observed a small increase in cohort number at ~40–60h with intermediate Treg ratios (as represented by a slight shift in the cohort plot to the right in 1:16, 1:8, 1:4), revealing a small effect on promoting survival. This explains the increased cell numbers seen in Figure 1C at this time. As undivided cells were not affected by Tregs (Figure 1B), this unexpected survival-enhancing effect of intermediate ratios of Tregs can be ascribed to the activated dividing-cell population.

### Regulatory T cells subtract from the mean division destiny reached by activated effector T cells in a dose-dependent manner

Late in the culture (after 70 h), there is a clear dose-dependent effect of Tregs on Teff cell number (Figure 1C), which represents the predominant suppressive effect of Tregs, and is the time at which *in vitro* Treg assays are typically measured. The number of times cells divide before they return to quiescence (division destiny) has recently been demonstrated as a critical component of T cell responses (20, 24). Division destiny is observed in cohort analysis as a plateau in the mean division number over time. We hypothesized that the suppressive effect of Tregs might be explained by regulation of division destiny or other features of cell division rate.

**Figure 2:**
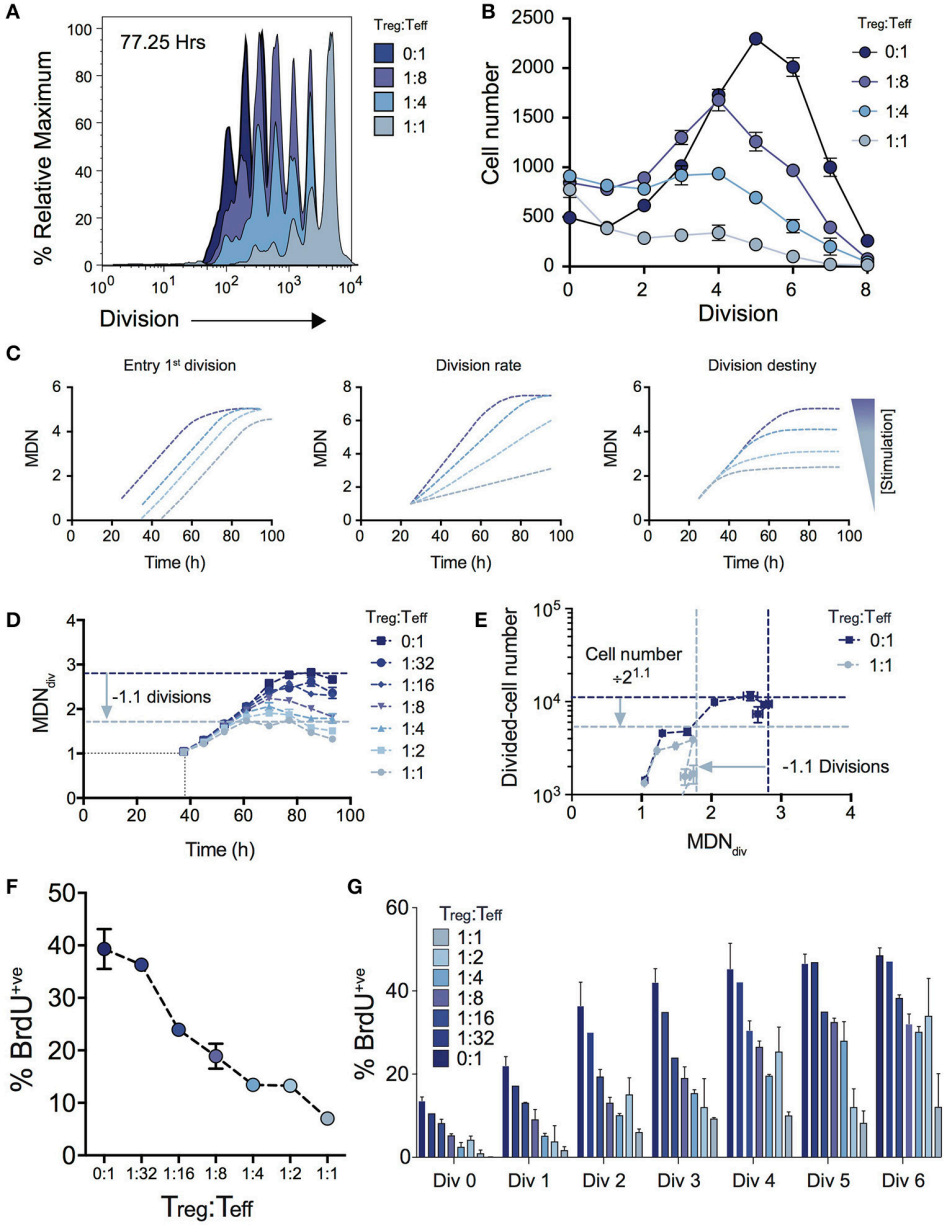
Using cohort analysis to dissect the effect of Tregs on Teff cell division. **(A)** Cell division profiles of Teff cells at 77.25 hours with varying ratios of Tregs. **(B)** Cell numbers per division at 77.25 h as determined by quantification to a known number of added beads. **(C)** Cohort plot examples illustrating how trends in graphs are altered by changes in mean time to 1st division, the subsequent division rate and division destiny, as labeled. MDN - mean division number. **(D)** Cohort analysis plot of Mean division number of divided Teff cells over time (cohort method, excluding undivided cells). **(E)** Divided Teff cell number (excluding undivided cells) vs. mean division number of divided cells (cohort method) in the presence and absence of Tregs. The darker horizontal and vertical dashed lines indicate division destiny in the absence of Tregs, the lighter dashed lines indicate the reduction in division destiny at the maximum ratio of Tregs:Teffs (1:1), and the predicted reduction in total live cell number. BrdU incorporation at 63 h as a function of Treg:Teff ratio for the total culture **(F)** and per division basis **(G)** during a 2 h BrdU pulse. Data shown are mean +/– SEM of triplicate samples. One representative data set from three independent experiments is shown.

Figure 2A shows the effect on cell division for varying Treg ratios illustrating a progressive reduction in T cell proliferation as Treg numbers are increased. The consequence of this effect on expansion of cell numbers is highlighted by the significant effect on the number of cells in each division (Figure 2B). Given the absence of Treg induced cell death (Figure 1D), we used the cohort method to investigate other potential kinetic influences that could explain the reduced division progression associated with increasing Treg numbers, namely time to first division, subsequent division rate (after first division) and division destiny. Figure 2C illustrates how changes to these distinct proliferation parameters (i.e., time to first division, division rate or division destiny) will affect cohort plots of mean division number vs. time (20, 21, 24, 27, 28, 31, 32). Figure 2D shows the effect of Treg co-culture on MDN_div_, the calculated mean division number of cells that have entered into division (i.e., excluding undivided cells) using the cohort method. This analysis demonstrates three important points of interest regarding the effect of Tregs on T cell stimulation: (1) Increasing the ratio of Tregs had no effect on the mean time taken for the Teff cell population to respond to stimulation and enter the first division (as indicated by the overlapping line for early divisions on the y-axis for all Treg ratios—Figure 2D). This is consistent with division tracking data from early time points in Figure 1A (37.50 h) shown with and without high Treg exposure. Here, no difference is observed in the first entry of responding cells into division; (2) The rate of division (the gradient of the mean division number vs. time curve) was unaffected by the presence of Tregs, but division destiny was reached earlier, consistent with a timed regulation of division destiny (24); and, (3) Increasing the ratio of Tregs reduced the maximum mean division number reached by Teff in a dose-dependent manner. Together, in the absence of a significant effect observed in all other parameters measured, this suggests that the predominant effect of Tregs is limiting the division potential of responding effector T cells.

To further demonstrate the quantitative effect of regulation of division destiny, we calculated the expected reduction in cell number that can be attributed to the diminished division destiny. This calculation is illustrated in Figure 2E. We compared proliferation in the absence of Tregs (ratio 0:1), to the highest ratio of Tregs (1:1). The difference in mean division destiny (dark blue vs. light blue lines) was determined to be 1.1 (Figure 2D); thus the expected reduction in cell number is 2^1.1^ = 2.14. We compared the number of divided cells vs. mean division number of divided cells (Figure 2E). Here, the dark blue horizontal line indicates the peak response measured in the absence of Tregs, while the light blue horizontal line represents the predicted reduction in cell number. Strikingly, the vast majority of the effect of adding Tregs to stimulating T cell conditions can be explained by changes in division destiny alone.

To confirm the effect of Tregs on proliferation, we investigated cell cycle turnover by measuring BrdU incorporation. As expected, the presence of Tregs reduced BrdU incorporation in a dose-dependent manner indicating fewer cells were actively dividing at higher Treg:Teff ratios at 63 h post stimulation when measured at either the total population (Figure 2F) or per division basis (Figure 2G). Thus, while consistent with *in vitro* Treg assays, our analyses provide further detail regarding suppressive mechanisms that regulate Teff kinetics.

### The quantitative effect of tregs on teff proliferation can be mimicked by known mechanisms of suppression

**Figure 3:**
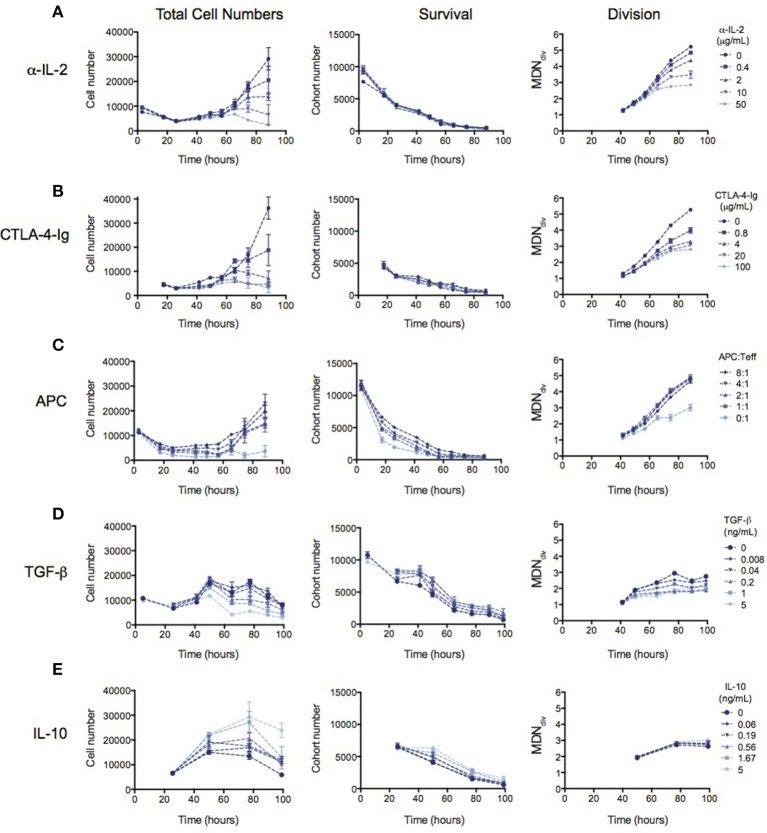
The kinetic effects of Treg suppression may be mimicked by some, but not all, known mechanism of Treg action. Teffs were stimulated with irradiated splenocytes (APCs) and anti-CD3. **(A)** Titration of an anti-IL-2 blocking antibody (S4B6)—total cell number (left panel), cohort number (middle panel) and mean division number of divided cells (MDN_div_, right panel). Titration of **(B)** CTLA4-Ig, **(C)** APC number, **(D)** TGF-β and **(E)** Titration of IL-10. Data shown are mean +/– SEM of triplicate samples. One representative data set from two independent experiments is shown.

Many mechanisms of suppression by Tregs have been demonstrated in a range of different *in vitro* and *in vivo* systems (16, 17). We therefore investigated whether the observed reduction in division destiny could be replicated by previously-studied mechanisms. In Figure 3, the effect of previously implicated mechanisms on the kinetics of Teff responses is investigated using the same quantitative assays outlined above. Total cell number (left panel), cohort number (survival—middle panel) and mean division number (Division analysis—right panel) is displayed for each experiment in order to illustrate effects on cell death and division destiny.

The availability of IL-2 has been shown to increase division destiny in a dose-dependent manner in T cells (20). Absorption of IL-2 by Tregs, and therefore reducing the access to free IL-2 has been described as a mechanism of Treg suppression (5–8). To mimic this effect, we added an anti-IL2 blocking antibody (S4B6) to cultures of Teffs stimulated with anti-CD3 and APCs (Figure 3A). Similar to the effect of Tregs, anti-IL-2 reduced division destiny without affecting cohort number. Next, we mimicked the effect of inhibition of co-stimulation, by adding CTLA4-Ig to cultures (Figure 3B). CTLA4-Ig binds to CD80 and CD86 and competitively blocks engagement of CD28 on T cells (33). Again, similar to the effect of Tregs, CTLA4-Ig did not affect cohort number but had a clear effect on reducing division destiny. There was also a small reduction in time to first division consistent with the effect of CD28 co-stimulation in the presence of IL-2 (29). By contrast, the number of APCs added to Teff cultures affected predominantly cohort number (Figure 3C). APC ratios between 1:1 and 8:1 did not appear to regulate division destiny. Thus, the APCs in this system appear to be important for survival of Teffs, through a mechanism that is not fully recapitulated by inhibition of IL-2 or co-stimulation.

Finally, we analyzed the effect of inhibitory cytokines, TGF-β and IL-10 (9, 10, 12–14). TGF-β modestly increased cohort number while reducing division destiny in a dose-dependent manner (Figure 3D, middle and right panels). The net effect of TGF-β was suppressive, as indicated by a decrease in total cell number (Figure 3D, left panel). This suppressive effect is interesting and unusual, as previous studies have shown that the addition of cytokines or increasing the level of receptor stimulation leads to an increase in division destiny as opposed to the direct subtraction observed here (20). Similar to TGF-β, addition of IL-10 modestly increased cohort number, however there was no effect on division destiny (Figure 3E, middle and right panels). Therefore, the net effect of IL-10 was to increase total cell number (Figure 3E, left panel). Thus IL-10 was not directly suppressive in this *in vitro* system. While surprising, a similar lack of suppression has been previously reported using a quantitative *in vitro* CD8+ T cell system (20).

## Discussion

Our results demonstrate that the predominant effect of Tregs is on reducing the division destiny of effector T cells, rather than directly reducing survival or division rate. This finding underscores the importance of division destiny as a key mechanism regulating the T cell expansion in activating as well as suppressive conditions.

**Figure 4:**
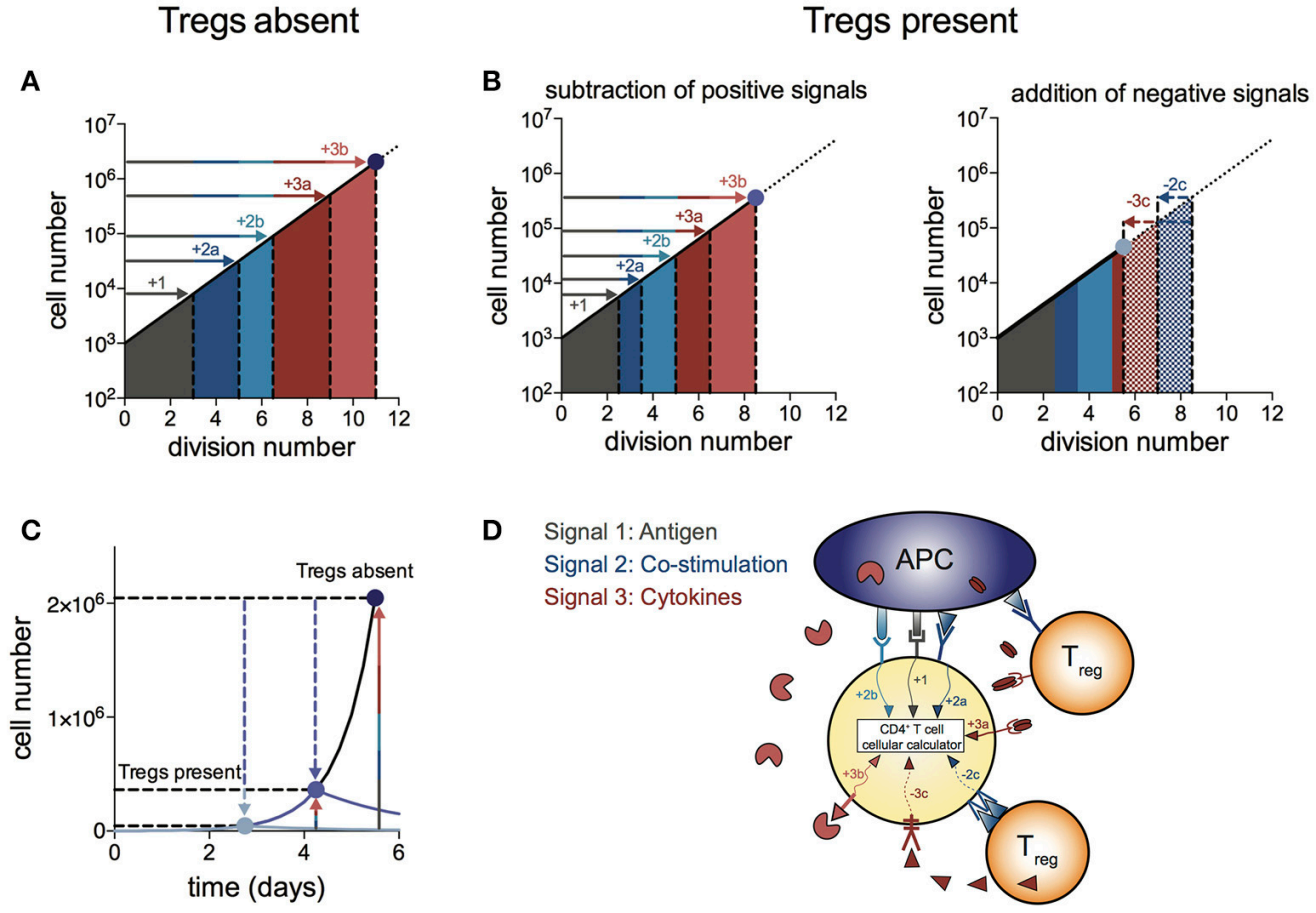
Log-dampener model of suppression of CD4^+^ effector T cell proliferation by Tregs. **(A)** In the absence of Tregs, signals from antigen (signal 1—gray), co-stimulation (signal 2—blue) and cytokines (signal 3—red) sum linearly according to the number of divisions contributed to division destiny by each signal, resulting in an exponential increase in the magnitude of the T cell response (20) (note the log scale on the y-axis). “a” and “b” refer to different types of co-stimulatory and cytokine signals contributing positively to the T cell response. **(B)** Tregs regulate division destiny by reducing positive signals (1, 2a and 3a) and by adding negative signals (2c and 3c). **(C)** Illustration of the effect of Tregs on the size of the effector T cell response (note the linear y-axis). The blue dots represent the peak of the T cell response in the absence of Tregs (dark blue), after subtracting of positive signals (medium blue) and after addition of negative signals (light blue). **(D)** Schematic showing different types of signals being integrated within the CD4^+^ effector T cell according to the rules of the “cellular calculus”.

We propose a “log-dampener” model of Treg suppression as illustrated in Figure 4. As shown in (20), contributions of antigen (signal 1), co-stimulation (signal 2) and cytokines (signal 3) to T cell division destiny can be summed linearly to predict the magnitude of the response (Figure 4A), thus providing a quantitative basis for classic two- and three-signal theories (34–37). Figure 4B shows the effect of Tregs in removing or reducing some of the positive signals (left panel), as well as supplying negative signals (right panel). Examples of reducing positive signals include CTLA4 binding to CD80/86 and inhibition of IL-2 by absorption or decreased production. Tregs also reduce CD80/86 directly on APCs to regulate co-stimulation strength (2, 38–40). Examples of addition of negative signals include TGF-β produced by Tregs acting on effector T cells. We were not able to show a similar mechanism for IL-10 in the *in vitro* system, suggesting a more complex mechanism of action to induce suppression *in vivo*, rather than a direct effect on the proliferation of effector T cells. Figure 4C illustrates the effect of removal of positive signals and addition of negative signals by Tregs on effector T cell numbers over time. As changes in division destiny translate to exponential effects on cell numbers, seemingly small perturbations can result in orders of magnitude difference in the peak number of T cells. Multiple pathways may sum independently to achieve suppression, and it is likely that the different pathways vary in their importance in different *in vivo* systems. Figure 4D illustrates the log-dampener model in schematic form. Our data highlights the dominant role of reducing division destiny in Treg action under these commonly employed culture conditions. It remains possible that other features might be targeted under different stimulation conditions (for example, antigen-specific T cells and dendritic cells). We anticipate that our assay methods employed here can be adapted and will prove useful to dissect such alternative cell arrangements.

A corollary of this model is that the classic *in vitro* suppression assay (frequently used for studies of Treg function and mechanism), is finely tuned to demonstrate this suppressive effect. The difference in division destiny of dividing Teffs between no Tregs and an equal ratio of Tregs was only slightly more than a single division cycle (Figure 1). The classic assay of tritiated thymidine incorporation on day 3 cannot distinguish between direct induction of cell death, slowing proliferation rate or reduction in division destiny. Studies of Treg function following genetic manipulation may benefit from using these quantitative methods to study the full kinetics, to assist with drawing conclusions as to the effect of the manipulation on function. Further studies with similar quantitative methods investigating different levels of TCR stimulation/affinity or varied sources of APC may be useful for dissecting whether division destiny is a universal mechanism that is affected by Treg regardless of culture conditions. Our study also indicated the surprising result that at some ratios Tregs enhanced net cell numbers by promoting survival of effector T cells. Two cytokines produced by Treg, TGF-β and IL-10 also promoted survival, potentially explaining this result. Thus, it appears the net outcome of Treg interaction with Teff results from combinations of positive effects on survival and negative influences on division destiny.

In conclusion, our results demonstrate that the complex and multifactorial suppressive effect of Tregs is amenable to study using rigorous quantitative techniques. The many known mechanisms of suppression either remove positive signals or supply negative signals, and combinations act on division destiny according to a simple cellular calculus – addition or subtraction from division destiny. Thus, by reducing division destiny of effector T cells, Tregs act as a “log-dampener” on the magnitude of the Teff response. The net effect is that small changes in division destiny induced by Tregs can have large effects on the peak size of the effector T cell response, with consequences for achieving the balance between protective immunity and tolerance of self.

## Author contributions

MD and EH designed and conducted experiments and wrote the manuscript. SH and JM contributed to data analysis, interpretation, and wrote the manuscript. AK performed mathematical modeling and data analysis. PH designed and supervised experiments and wrote the manuscript.

### Conflict of interest statement

The authors declare that the research was conducted in the absence of any commercial or financial relationships that could be construed as a potential conflict of interest. The reviewer DS and handling Editor declared their shared affiliation.
